# Major factors influencing student engagement in Ethiopian higher education institutions: Evidence from one institution

**DOI:** 10.1371/journal.pone.0318731

**Published:** 2025-02-06

**Authors:** Mekonnen Haile Faro, Tariku Sime Gutu, Adula Bekele Hunde

**Affiliations:** 1 Department of Educational Technology and Information Management, Mattu University, Metu, Ethiopia; 2 Departments of Teacher Education and Curriculum Studies, Jimma University, Jimma, Ethiopia; 3 Department of Teacher Education and School Research, University of Oslo, Oslo, Norway; Ahvaz Jundishapur University: Ahvaz Jondishapour University of Medical Sciences, ISLAMIC REPUBLIC OF IRAN

## Abstract

This study investigates factors influencing student engagement in Ethiopian higher education, focusing on self-efficacy, teacher support, and technology support at Mattu University. A cross-sectional survey design was employed; involving 620 undergraduate students selected using stratified sampling. Data were collected through structured questionnaires and analyzed using Structural Equation Modeling (SEM) to assess the impact of these variables on student engagement. The findings reveal that teacher support, technology support, and self-efficacy collectively explain 69% of the variance in student engagement, with teacher support identified as the strongest predictor. The results highlight the importance of supportive teacher-student relationships, accessible digital resources, and students’ confidence in their academic abilities for fostering engagement. The study underscores the need for improved teacher preparation, diverse instructional strategies, and enhanced access to digital resources. These findings offer practical recommendations for policymakers and educational institutions in Ethiopia to create more engaging learning environments, ultimately enhancing academic success and retention in higher education.

## Introduction

Student engagement is a critical strategy for achieving educational objectives [1, 2]. It plays a central role in effective learning, requiring students to actively interact with peers, teachers, and learning materials to enhance their learning experiences [3]. Engagement in higher education is crucial for enriching students’ learning experiences and effectively preparing them for future careers [4].

Engaging students can yield a range of both short- and long-term effects, encompassing positive social and academic outcomes. In the short term, social benefits include enhanced relationships, a sense of competence, belonging, and overall satisfaction. Academic benefits may involve improved knowledge, increased interest, greater confidence, and better study habits. Over the long term, academic advantages of student engagement include higher retention rates, greater success in the workplace, and a commitment to lifelong learning. Socially, the long-term outcomes can include improved citizenship, personal development, and greater institutional involvement [5].

The concept of "student engagement" remains complex and lacks a universally accepted definition. Scholars offer various interpretations of the term. Astin (1984) defined “student involvement” as the physical and psychological energy students invest in their college experience [6]. Although his theory focuses primarily on the behavioral aspect, it contributes significantly to the study of student engagement [7]. Krause (2005) described engagement as the time, energy, and resources students dedicate to activities that enhance their learning experience [4]. Similarly, some scholars define engagement based on students’ completion of tasks and assignments for class [8].

Other scholars have expanded the concept to include multiple dimensions. London et al. (2007) define engagement as encompassing academic investment, motivation, commitment, psychological connection, comfort, and a sense of belonging [9]. Schaufeli et al. (2002) view engagement as a multifaceted construct that includes effort, resilience, persistence, passion, inspiration, pride, and involvement in academic learning activities [10]. Bowden et al. (2019) defined student engagement in higher education as the positive investments—social, cognitive, emotional, and behavioral—that students make in their institution and its members [11]. Fredricks et al. (2004) proposed a holistic definition, emphasizing three types of engagement: behavioral, emotional, and cognitive, with each type including specific indicators such as participation, effort, attention, interest, and problem-solving skills [12].

The various definitions of student engagement, presented across different times and contexts, are summarized in Table 1, highlighting that the construct lacks a single, specific definition (Table 1).

**Table 1:** Definitions of student engagement by various authors.

Author/s	Definition of Student Engagement
[6]	Defined as the physical and psychological energy students invest in the college experience.
[13]	Engagement represents students’ emotional and behavioral participation in classroom settings, crucial for motivation.
[10]	Engagement as a multifaceted construct including effort, resiliency, passion, and pride, focused on academic success.
[12]	Defined as a multidimensional concept involving behavioral, emotional, and cognitive aspects of engagement, each with specific indicators.
[4]	Defined engagement as the time, energy, and resources that students invest in learning-enhancing activities.
[9]	Engagement includes motivation, commitment, psychological connection, and a sense of belonging
[14]	Engagement encompasses academic behaviors, attitudes, and motivation that influence student success in school settings.
[15]	Engagement is the time and effort students dedicate to educational activities in alignment with institutional expectations.
[16]	Student engagement is the investment in, and commitment to, learning activities within academic environments.
[17]	A student’s positive social, cognitive, emotional, and behavioral investments in engaging with university peers, staff, and the institution itself.
[18]	A state of emotional, social, and intellectual readiness to learn, driven by curiosity and active participation.

The definitions provided highlight the diverse and multidimensional nature of student engagement, encompassing physical, emotional, cognitive, social, and behavioral dimensions. While some emphasize the energy and time invested in academic activities, others focus on motivation, resilience, and a sense of belonging within educational environments. This variety underscores the complexity of the construct, with each perspective contributing unique insights into its role in student success.

The global economy increasingly demands qualified professionals, prompting academic institutions and scholars to prioritize effective student engagement [19]. Despite this, research indicates a decline in students’ enthusiasm and motivation for learning [12]. Numerous studies have explored factors that enhance or hinder engagement. For example, a meta-analysis of 93,188 participants by Li and Xue (2023) identified key factors: positive emotions, active learning behaviors, strong teacher-student relationships, and adequate learning resources promote engagement, while negative behaviors and lack of environmental support hinder it [20]. Additionally, research highlights that technology-enriched classrooms, supportive learning environments, respectful peer and teacher interactions, and collaborative learning cultures—where teachers and students learn together—are essential for fostering engagement [21]. Axelson and Flick (2010) emphasize that engagement emerges from a well-designed learning environment, where students are actively involved and teachers provide consistent support to sustain motivation and participation [22]. These findings underline the need for deliberate efforts to create dynamic and inclusive learning spaces that promote active student involvement.

Furthermore, research shows that both internal and external factors in higher education can influence student engagement. External factors relate to teachers and institutions, while internal factors are specific to students [23]. Student-related factors include academic self-efficacy, motivation, satisfaction, performance, academic background, and values. Teacher-related factors involve teaching style, behavior, and the teacher-student relationship. Institutional factors encompass resources like libraries, laboratories, social services, technology, and organizational policies [24].

In summary, student engagement in higher education is shaped by a complex interplay of internal factors, such as self-efficacy and motivation, and external factors, including teacher behaviors and institutional resources, which together can either support or hinder student engagement.

This study examines the influence of three key factors—student self-efficacy, teacher support, and technology support—on student engagement in academic matters, focusing on its three dimensions: behavioral, emotional, and cognitive, using different indicators for each [12].

### Rationales of the study

Student engagement is critical to academic success and holistic learning. Globally, challenges such as declining motivation, ineffective teaching practices, and resource limitations persist, particularly in developing countries like Ethiopia, where traditional teacher-centered pedagogies dominate and digital technology integration remains minimal [12, 23, 25–27]. Mattu University, as a third-generation institution, reflects these broader challenges in Ethiopian higher education. This study investigates factors influencing engagement—specifically self-efficacy, teacher support, and technology support—within the context of Mattu University [28]. By addressing these factors, the research contributes to understanding how engagement can be improved in resource-limited settings while supporting Ethiopia’s educational development goals [27].

Mattu University’s reliance on traditional teaching methods presents a distinctive context for studying engagement dynamics. Like other Ethiopian universities, it faces significant challenges, including limited resources and inadequate technological support [26]. Student self-efficacy, a key driver of engagement, is often undermined by negative academic self-perceptions and insufficient teacher support [29, 30]. Additionally, resource constraints hinder effective technology use, creating a gap between students’ expectations and available tools. This study investigates the individual and combined effects of self-efficacy, teacher support, and technology support on student engagement, providing insights to enhance institutional practices and promote a more engaging and equitable learning environment.

Addressing these three factors collectively is essential, as they are individually linked to student engagement but seldom studied in combination within Ethiopian higher education [24, 31]. The low engagement levels across Ethiopian universities, including Mattu University, highlight the urgency of exploring effective strategies [32, 33]. By focusing on these factors, this study bridges research gaps and provides actionable recommendations for enhancing engagement, responding to national policies advocating for digital transformation and student-centered learning. Ultimately, the findings can inform both institutional practices at Mattu University and broader strategies for engagement in similar contexts.

#### Student self-efficacy

Student engagement is strongly influenced by self-efficacy; students who believe in their abilities are more likely to set high goals, persist, and actively participate in academic tasks [34, 35]. Academic self-efficacy reflects confidence in completing tasks, fostering interest, and promoting persistence and success [36]. It is a key predictor of engagement, as students with high self-efficacy are more effective in classroom activities and maintain long-term academic involvement [37, 38]. High self-efficacy manifests in behaviors like asking questions and tackling challenging coursework, while low self-efficacy leads to reduced motivation and performance [39]. This can result in test anxiety and avoidance of difficult tasks, ultimately reducing engagement and academic performance [40–42]. Addressing low self-efficacy is crucial for fostering resilience and sustained engagement [43]. In summary, high self-efficacy directly supports student engagement by enhancing confidence and motivation.

In the Ethiopian higher education context, low self-efficacy contributes to academic dishonesty, such as cheating and plagiarism, undermining the integrity of learning [27, 44]. Students with low confidence may resort to dishonest practices due to feelings of inadequacy [45]. The widespread use of mobile phones and the internet further facilitates these behaviors [44]. Research indicates that improving self-efficacy can reduce academic misconduct, as confident students are less likely to cheat [46, 47]. Innovative, student-centered interventions—like interactive teaching and personalized feedback—can help boost self-efficacy, motivating students and improving engagement in academic work [29].

#### Teacher support

Teacher support is critical for enhancing student engagement across higher education contexts. Positive teacher-student relationships and effective communication significantly impact student motivation and resilience [48, 49]. Teachers who utilize varied teaching techniques and encourage critical thinking can foster deeper engagement with content, promoting responsibility and problem-solving skills [50, 51]. Supportive teaching practices enhance intrinsic motivation and contribute to sustained academic involvement [52, 53].

In Ethiopia, the potential of teacher support is often hindered by traditional, lecture-based methods that limit engagement [26]. Despite policies advocating for student-centered approaches, many instructors continue to use outdated instructional practices, which reduce student motivation [54]. Research shows that inadequate teacher support and lack of innovative teaching methods contribute to low engagement and poor academic performance in Ethiopian universities [33, 55]. To address these issues, incorporating comprehensive support systems, such as personalized feedback and interactive teaching, is crucial for fostering an engaging learning environment and improving academic success [56].

#### Technology support

Technology support significantly impacts student engagement in higher education. Digital technologies promote active learning by enabling diverse teaching methods, providing individual access to resources, and facilitating communication with peers and instructors [57, 58]. These technologies encourage student independence and responsibility while offering accessible learning environments for students with specific needs [59, 60]. By fostering engagement through interactive digital platforms, students become more motivated and involved in their studies, aligning with the needs of today’s "digital natives" [61, 62].

In Ethiopia, while the integration of digital technologies into higher education is progressing, challenges remain. Limited internet access, insufficient ICT infrastructure, and a lack of skilled personnel continue to hinder full integration, despite strong policy support and government initiatives to enhance digital infrastructure [63].

**Fig 1:**
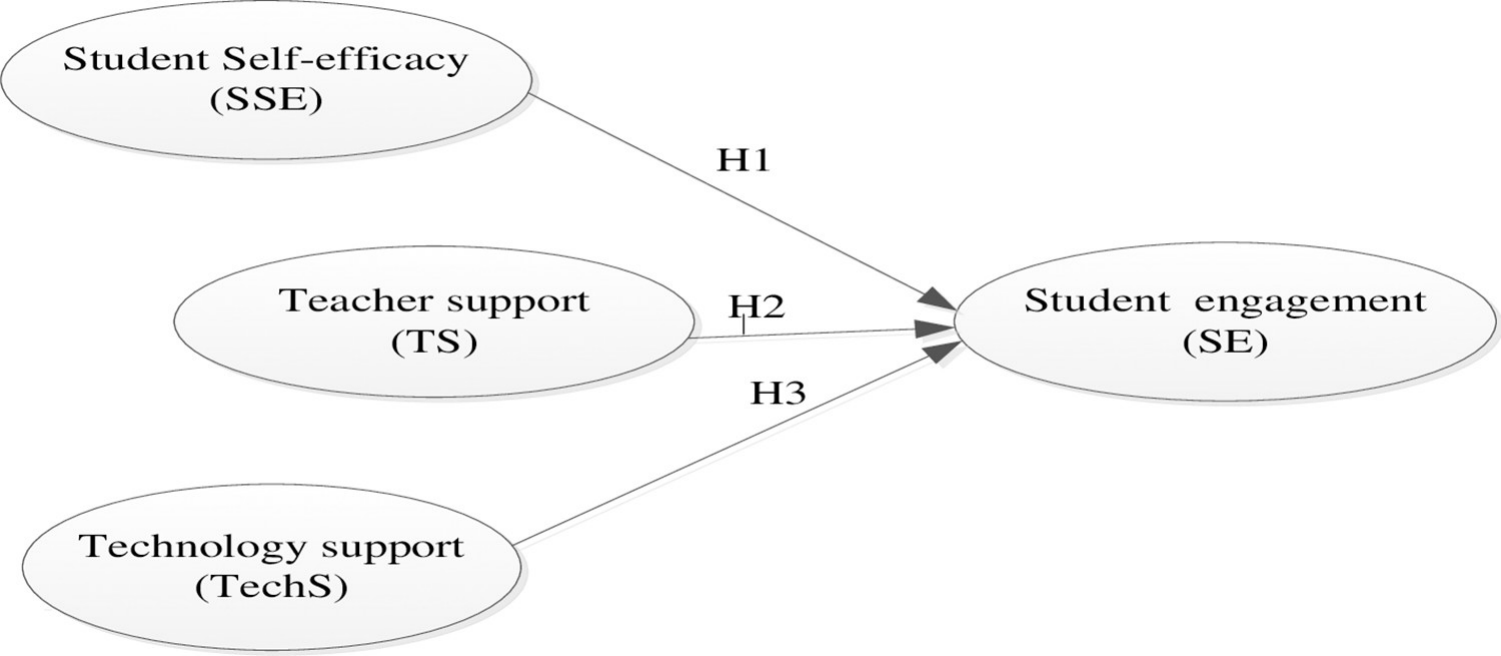
Conceptual framework of the model.

Thus, this study examines how self-efficacy, teacher support, and technology support influence student engagement at Mattu University, both individually and collectively. By exploring these interconnected factors, it aims to provide actionable insights to improve educational practices within Ethiopia’s higher education landscape. To achieve this purpose, the following hypotheses were proposed based on the study’s rationales (Fig 1).

1. Hypothesis 1(H1): Student self-efficacy has a direct positive significant influence on student engagement at Mattu University, Ethiopia.
2. Hypothesis 2(H2): Teacher support has a direct positive significant influence on student engagement at Mattu University, Ethiopia.
3. Hypothesis 3(H3): Technology support has a direct positive significant influence on student engagement at Mattu University, Ethiopia.
4. Hypothesis 4(H4): Self-efficacy, teacher support, and technology support have a direct, positive, significant combined influence on student engagement at Mattu University, Ethiopia.

### Theoretical framework of the study

Astin’s (1984) Theory of Student Involvement emphasizes that students’ physical and psychological investment in academic activities is fundamental to their learning and development [6]. Fredricks, Blumenfeld, and Paris’s (2004) model explains student engagement in three dimensions: behavioral, emotional, and cognitive. This framework offers a complete view of how students interact with their learning environment [12]. The combined effects of student self-efficacy, teacher support, and technology support play a critical role in shaping student engagement in higher education. Self-efficacy, as posited by Bandura (1997), influences students’ motivation and persistence, which in turn enhances their academic engagement [64]. When students believe in their capabilities, they are [65], more likely to actively participate in learning activities. Teacher support fosters a positive learning environment where students feel emotionally and academically supported, which is essential for engagement [51]. Furthermore, technology support, as outlined in the Unified Theory of Acceptance and Use of Technology (UTAUT2) [66], emphasizes the importance of providing students with the tools and resources necessary to engage with digital learning platforms effectively. Recent studies show that self-efficacy, teacher support and technology support work together to improve students’ behavior, emotions, and cognitive engagement. This combined effect leads to better academic engagement [67, 68]. This integrated framework highlights the integrated impact of these factors on student engagement in higher education in Ethiopian contexts, particularly at Mattu University.

## Methods and materials

### Research design

The study utilized Cross-sectional survey design. Cross-sectional survey design is a popular method in education, collecting data at a single point in time to examine current attitudes, beliefs, opinions, or practices [69]. Thus, the design was used to collect data on the level of student engagement and three major influencing factors, including technology support, teacher support, and self-efficacy.

### Participants

The study employed stratified random sampling combined with the Yamane formula to ensure proportional representation across programs and academic years. Stratified sampling is highly effective in ensuring proportional representation across diverse subgroups, reducing sampling bias, and enhancing generalizability in educational research. This approach is particularly valuable when characteristics like academic programs or year levels significantly influence outcomes, allowing for more precise subgroup comparisons and robust findings [70]. The university offers 45 undergraduate programs within six colleges, spanning one- to five-year cohorts. From these, 15 programs (approximately 30%) were randomly selected. First-year students were excluded, as their limited academic exposure (only one month of classes) was insufficient for the study’s requirements. For the remaining cohorts, third- and fourth-year students were selected using a lottery method. This method reflects the diversity of the student population and aligns with established educational research practices, where the Yamane formula and stratified sampling are valued for their accuracy and reliability in generating generalizable results [71, 72].

The sample size was determined using the Yamane formula with a 5% margin of error (e = 0.05), a standard approach in social research. Calculations were conducted separately for each of the 15 selected programs. This yielded a total sample size of 620 students, including 294 third-year and 326 fourth-year participants. The Yamane formula is widely recognized in social sciences and educational research for its practicality in handling large datasets and ensuring the generalizability of findings [73].

### Instruments and data collection

The constructs—student self-efficacy (SSE), teacher support (TS), technology support TechS), and student engagement (SE)—were treated as defined in Table 2. The table conceptualizes key constructs influencing student engagement, providing definitions and supporting references to clarify their role in academic settings. Student self-efficacy (SSE) reflects students’ confidence in completing tasks and achieving goals. Teacher support (TS) highlights the role of academic encouragement from instructors, while technology support (TechS) emphasizes access to digital tools for learning. Finally, student engagement (SE) is defined as a multidimensional construct, encompassing behavioral, emotional, and cognitive participation in learning activities.

**Table 2:** Conceptualizations of the key constructs.

Construct	Conceptualization	References
Student self-efficacy (SSE)	It represents the belief that students have in their ability To finish academic tasks and achieve specific goals.	[38, 74, 75]
Teacher Support (TS)	It refers to academic support that teachers provide to their students in order to encourage meaningful academic engagement.	[50, 51, 76]
Technology Support (TechS)	This underscores the significance of guaranteeing access to digital technologies in order to effectively utilize digital resources.	[77, 78]
Student Engagement (SE)	Student engagement is a multidimensional concept encompassing behavioral, emotional, and cognitive aspects, each characterized by distinct indicators such as participation, interest, effort, and critical thinking.	[12]

This study employed data collection tools developed from various sources, consisting of four main components: student self-efficacy (SSE), teacher support (TS), technology support (TechS), and student engagement (SE), as outlined in Table 3. This table outlines the items for Student Self-Efficacy (SSE), Teacher Support (TS), and Technology Support (TechS), designed to assess the impact of these key factors on student engagement in the learning environment. The instruments for Student Engagement (SE) were specifically developed to evaluate the current state of student engagement and its relationship with these three influential factors.

**Table 3:** Measurement constructs, sample items, and sources for assessing student engagement.

Constructs	Examples of the measurement items	Sources
SSE	I have confidence in my ability to comprehend difficult course material. With my fellow students, I usually have academic discussions.	[79, 80]
TS	Instructors integrate collaboration among learners into their lessons I feel at ease discussing ideas with my teachers	[81–83]
TechS	My institution has equipped digital library I have access to learning materials at all times and places.	[77, 84]
SE	I actively participate in class discussions (behavioral aspect). I collaborate with other students, and we all gain knowledge from one another (emotional aspect). I try to put new concepts I’ve learned into my own words (Cognitive aspect)	[12, 17, 18]

Establishing both face and content validity is crucial to ensure the accuracy and relevance of research tools. In this study, face validity, which assesses the clarity and appropriateness of items as perceived by participants, was verified through reviews by language and psychology experts, with revisions made to enhance clarity [85].

Content validity, assessing how well items represent the intended construct, was measured using the Content Validity Index (CVI). Seven experts from diverse academic backgrounds evaluated items related to student self-efficacy, teacher support, technology support, and student engagement on a 4-point scale: not relevant, slightly relevant, fairly relevant, or highly relevant. To be retained, an item’s CVI had to exceed 0.83 [86, 87]. The experts’ ratings produced CVI scores ranging from 0.86 to 1, confirming all items met the threshold (Table 4). Nonetheless, some items were refined based on expert feedback. These findings demonstrate that the instruments are valid, conceptually aligned, and suitable for the study context.

**Table 4:** Content Validity Index (CVI) ratings by experts.

Expert Number	Average Rating (Out of 4)	Content Validity Index (CVI)
Expert 1	3.9	0.90
Expert 2	3.8	0.87
Expert 3	3.7	0.86
Expert 4	4.0	1.00
Expert 5	3.8	0.88
Expert 6	3.9	0.90
Expert 7	3.9	0.89
Overall CVI	3.9	0.88

Rating Scale: 1 = Not Relevant, 2 = Slightly Relevant, 3 = Fairly Relevant, 4 = Highly Relevant

Pilot testing is essential for evaluating the clarity, relevance, and functionality of instruments in psychometric studies. A sample size of 100 to 150 participants is considered appropriate, ensuring reliable results and reducing biases associated with smaller samples. For instance, Kennedy (2022) highlighted that sample sizes below 100 are less reliable, whereas larger samples improve reliability [88]. Cronbach’s alpha is a widely used measure of internal consistency, with values above 0.70 considered acceptable and those exceeding 0.90 indicating excellent reliability [89]. Pilot testing refines instruments by eliminating ambiguities and strengthening validity, as emphasized by Polit and Beck (2006) and Chikezie and Eme (2023) [86, 87].

In this study, pilot testing involved 150 students from a college located on a separate campus, excluding the target colleges for the main study. Five-point Likert scales were employed, ranging from "strongly disagree" (1) to "strongly agree" (5), as they are effective for capturing respondents’ experiences and opinions [90]. The calculated Cronbach’s alpha indicated strong reliability for each construct: student engagement (0.91), student self-efficacy (0.89), teacher support (0.92), and technology support (0.93) (Table 5). Five items were removed, and seven were rephrased due to frequent omissions by participants during the rating process.

**Table 5:** Reliability analysis of constructs with item count and Cronbach’s alpha.

Constructs	Number of Items	Cronbach’s alpha
Student Self-Efficacy (SSE)	10	0.89
Teacher Support (TS)	10	0.92
Technology support (TechS)	7	0.93
Student Engagement (SE)	30	0.91

#### Data analysis

Structural equation modeling (SEM) is utilized to verify the proposed framework, identifying latent attribute-observed indicator correlations, analyzing intricate linkages, and providing causal explanations [91]. The measurement model and the structural model were used in two steps of structural equation modeling (SEM) to examine the data. With SPSS Amos 23, confirmatory factor analysis (CFA) was used to test the proposed measurement model. Absolute fit 2, df, comparative fit index (CFI), goodness-of-fit index (GFI), Tucker-Lewis index (TLI), standardized root mean square residual (SRMR), and root mean square error of approximation (RMSEA) were used to measure the goodness of fit [92]. The test relied on alternative fit indices for all model fit evaluations because the chi-square value is sensitive to a large sample size [90, 92].

### Ethical consideration

Jimma University granted permission for the investigation, with ethics approval from the College of Education and Behavioral Science Institutional Review Board (IRB), ensuring compliance with exemption requirements. Next, we requested and received permission from Mattu University to contact the concerned colleges and departments of the university. Thirdly, we contacted colleges and departments to get the sampled students. The study involved classroom discussions with each discipline student in the classroom to obtain written consent. Participants were assured of free and informed choice, the freedom to withdraw at any time, and confidentiality. Following the brief discussions, they signed a consent form agreeing to fill out the questionnaires, and after their signature, the questionnaires were distributed. Participants’ anonymity and confidentiality were rigorously safeguarded throughout the study. Identifiable information was neither collected nor linked to responses, ensuring that all data remained anonymous [72].

## Results

### Descriptive statistics

Before analyzing the data, a normality test was conducted to assess whether the data followed a normal distribution. Skewness and kurtosis values were examined, with skewness values between +1 and -1 indicating symmetry and kurtosis values less than 2 suggesting a mesokurtic distribution, which aligns with the assumption of normality. To determine whether the data could be analyzed, a missing value analysis was also carried out [90]. In this study, no missing data were found, and the data distributions were symmetric and mesokurtic, as indicated by the skewness and kurtosis values (Table 6). Therefore, the data meet the assumptions for normality and can be analyzed further.

**Table 6:** Summary of skewness, kurtosis, symmetry, and normality for measured variables.

Variable	Skewness	Kurtosis	Symmetry	Normality
Student Self-Efficacy	-0.55	1.25	Symmetry	Normal
Teacher Support	0.33	1.13	Symmetry	Normal
Technology Support	-0.52	1.12	Symmetry	Normal
Student Engagement	0.31	1.15	Symmetry	Normal

#### Demographic characteristics of participants

The proportions of female and male students were 27% (n = 168) and 73% (n = 452), respectively. Of the individuals involved, 294 students (47.4%) and 326 students (52.6%) were selected from third-year and fourth-year batches, respectively (Table 7).

**Table 7:** Demographic characteristics of participants.

Demographic Category	Count (n)	Proportion (%)
Gender		
Female	168	0.27
Male	452	0.73
Year of Study		
Third Year	294	0.474
Fourth Year	326	0.526
Total	620	100

This table shows the gender and academic year distribution of participants. It highlights the proportional representation of male and female students and the inclusion of third- and fourth-year students. These details ensure the findings are generalized and provide insights into engagement across diverse groups at Mattu University.

#### Latent variable scores

On a scale of 1 to 5, the latent variable student self-efficacy (SSE) had item means ranging from 2.64 with a standard deviation of 0.69 ("I frequently ask my teachers for clarification when something is unclear") to 2.68 with a standard deviation of 0.68 ("When my friends ask me for academic assistance, I am confident that I can help them"). For latent variable teacher support (TS) on a scale of 1 to 5, item means varied from 2.65 with the standard deviation of (" Teachers offer opportunities for in-depth understanding, analysis, critical thinking, and problem solving") to 2.7 with the standard deviation of 0.71 ("I feel at ease discussing ideas with my instructors").

The latent variable technology support (TechS) had item means ranging from 2.65 with a standard deviation of 0.74 ("My institution provides students with adequate internet and ICT facilities") to 2.7 with a standard deviation of.73 ("My institution’s library services are adequate to satisfy my needs") on a scale from 1 to 5.

Student engagement (SE) item means ranged from 2.63 with a standard deviation of 0.73 ("I participate in group projects with classmates throughout class time") to 2.7 with a standard deviation of 0.73 ("I am energized by the activities we undertake in the classroom"). The participants assessed each item adversely, as seen by the descriptive data, where the means of the items were less than 3.0. A standard deviation between 0.69 and 0.73 indicates that the scores for each variable are relatively consistent, with most values falling within one standard deviation of the mean (Table 8). This table presents the mean scores and standard deviations for the key variables of student self-efficacy, teacher support, technology support, and student engagement, indicating the overall levels of these constructs among participants.

**Table 8:** Summary of latent variable scores with standard deviations.

Variable	Mean Score	Standard Deviation
Student Self-Efficacy (SSE)	2.66	0.69
Teacher Support (TS)	2.68	0.70
Technology Support (TechS)	2.67	0.73
Student Engagement (SE)	2.63	0.73

A smaller standard deviation reflects greater concentration of the data around the mean, suggesting that participants’ scores for these latent variables show low variability and are generally close to the average. These findings provide a basis for understanding the relative levels of these constructs and their potential influence on student engagement at Mattu University.

### Structural equation modeling (SEM)

The suggested model was created based on a literature study, and its approach was confirmatory in nature—that is, it was evaluated to see if the data fit the model [93]. Both the structural level and the measurement level were examined in the analysis of structural modeling [94].

#### Measurement model

Model fit statistics are crucial for evaluating how well a proposed model aligns with observed data, ensuring its validity for interpretation. These statistics involve several indices, each assessing different aspects of model fit. The Chi-square (χ^2^) test, paired with degrees of freedom (DF), measures the overall discrepancy between observed and predicted data, with a χ^2^/df ratio below 3 indicating an acceptable fit. The Comparative Fit Index (CFI) and Tucker-Lewis Index (TLI) compare the proposed model to a baseline model, with values above 0.90 representing an excellent fit. The Root Mean Square Error of Approximation (RMSEA) assesses error per degree of freedom, where values below 0.08 reflect a strong fit. The Goodness of Fit Index (GFI) evaluates the proportion of variance explained by the model, with a threshold of 0.90 indicating good alignment. Lastly, the Standardized Root Mean Square Residual (SRMR) quantifies the average residual differences between observed and predicted correlations, with values below 0.08 confirming a strong fit. Collectively, these indices offer a comprehensive assessment of the model’s quality and alignment with the data [92] (Table 9). This table presents the fit indices for the model, indicating that all indices (χ^2^, CFI, TLI, RMSEA, GFI, and SRMR) meet or exceed the recommended thresholds, demonstrating a good fit between the proposed model and the observed data.

**Table 9:** Measurement model fit statistics.

Fit Indices	Goodness of fit	Model	Fit Indices	Goodness	Model
				of fit	
**χ2**		580.429	CFI	≥.90	0.97
**Df**		246	TLI	≥.90	0.97
**χ2/df**	<3	2.4	RMSEA	< .08	0.047
**GFI**	≥.90	0.93	SRMR	< .08	0.012

Specifically, the fit indices provided validate the model’s alignment with the observed data. The Chi-square **(χ^2^)** value of 580.429 with 246 degrees of freedom and a χ^2^/df ratio of 2.4 indicates an acceptable model fit, as values below 3 are considered satisfactory for large sample sizes [92]. The Comparative Fit Index (CFI) and Tucker-Lewis Index (TLI) both exceed the threshold of 0.90, with values of 0.97, demonstrating excellent comparative fit. Similarly, the Goodness of Fit Index (GFI) of 0.93 surpasses the cutoff, reflecting strong absolute fit. Additionally, the Root Mean Square Error of Approximation (RMSEA)of 0.047 and Standardized Root Mean Square Residual (SRMR) of 0.012 are well below the thresholds of 0.08, indicating minimal error and good fit between the model and the data. These indices collectively confirm the model’s robustness, highlighting its validity for analyzing the relationships among variables in the study.

The model appears to match the data reasonably well, based on the combined findings of these fit indices. Every index either meets or surpasses the prescribed criteria, suggesting that the model accurately captures the underlying structure of the data. A thorough evaluation of the model’s fit is provided by the combination of absolute fit indices (χ2/df, GFI, RMSEA, SRMR) and comparative fit indices (CFI, TLI), which increases confidence in the model’s validity and suitability for the provided data.

#### Construct reliability and validity

Cronbach’s alpha (α), composite reliability (CR), average variance extracted (AVE), and discriminant validity were used to assess construct validity and reliability of the current data [95, 96]. High values for Composite Reliability (CR) across all dimensions are found in the construct analysis, indicating excellent internal consistency of the measured variables. For the SSE build, the CR values are 0.905, while for the AE construct, they are 0.933. The constructs’ reliability is confirmed by these results, which are higher than the widely recognized cutoff point of 0.7. Furthermore supporting the robustness of the model are the Average variation Extracted (AVE) values, which quantify the amount of variation collected by a construct relative to the variance caused by measurement error. The measurement model’s validity is further validated by the AVE values, which are satisfactory, with the SE construct’s lowest value being 0.755 (Table 10). This table presents the composite reliability (CR), average variance extracted (AVE), and Cronbach’s alpha values for each construct, demonstrating excellent internal consistency and validity for the study’s measures.

**Table 10:** Construct reliability and validity.

Constructs	CR	AVE	Cronbach’s alpha
**SSE**	0.905	0.772	0.904
**TS**	0.906	0.793	0.907
**TechS**	0.906	0.793	0.906
**SE**	0.933	0.755	0.933

Additionally, since all of the Cronbach’s alpha coefficients for each construct are higher than the suggested cutoff of 0.7, this further supports the model’s overall reliability. SSE and AE have coefficients ranging from 0.904 to 0.933, indicating a consistent measurement of the desired latent variable by each construct. Maintaining consistency is essential to guaranteeing that the gathered data can be confidently understood. When combined, these results imply that the study’s constructs have high levels of validity and reliability, which strengthens the validity of any further analyses and judgments made using the dataset (Table 10).

#### Discriminant validity of the measurement model

The correlation matrix demonstrates strong discriminant validity, indicating that constructs measuring different concepts are indeed distinct. The diagonal values for SSE, TS, TechS, and SE are greater than 0.80, indicating internal consistency. Inter-construct correlations show lower values, with the highest being 0.66 between SE and TS. For instance, the correlation between TechS and SE (0.64) is modest, further affirming that these constructs reflect different dimensions of measurement. Similarly, SSE, with a correlation of 0.65 with TS, and its lower correlations with other constructs bolster the case for discriminant validity. Overall, the data implies that each construct captures unique aspects of the theoretical framework, thus reinforcing the validity of the measurements used within this model [97] (Table 11). This table shows the correlation matrix for the constructs, with diagonal values representing internal consistency (Cronbach’s alpha) and off-diagonal values indicating the discriminant validity between the constructs, all of which meet the acceptable thresholds.

**Table 11:** Discriminant validity of the measurement model.

	SSE	TS	TechS	SE
**SSE**	**0.88**			
**TS**	0.65	**0.89**		
**TechS**	0.63	0.64	**0.89**	
**SE**	0.55	0.66	0.64	**0.85**

#### Structural model

The measurement model has been successfully tested, and the results reveal that the proposed research model matches the data well; nonetheless, the current section focuses on the structural model’s analysis. The purpose of this study was to examine the impacts of three suggested variables on student engagement.

The statistical research shows that self-efficacy, technology support and teacher support are the three primary factors that significantly positively correlate with student engagement. The three variables exhibit statistically significant impacts (p < .001), as indicated by their respective standardized coefficients of 0.254, 0.27, and 0.354. Self-efficacy, while substantial, has a relatively smaller effect on student engagement than technology support and teacher support. With an R-squared value of 0.69, the model demonstrates a significant ability to explain variance, with these three components accounting for 69% of the variance in student engagement (Table 12). Therefore, the table presents the standardized coefficients, standard errors (S.E.), t-values, and p-values for the hypothesized relationships, all of which are statistically significant.

**Table 12:** Path analysis results for hypothesized relationships and student engagement.

Hypothesized relationship	Standardized Coefficient	S.E.	T	P-value	Decision
**SSE->SE**	0.254	0.074	3.664	***	Supported
**TS->SE**	0.354	0.069	4.913	***	Supported
**TchS ->SE**	0.27	0.062	3.997	***	Supported
R- squared					
**Student engagement**	**0.69**				

Note.

***P< .001

These results emphasize the complexity of student engagement and the value of an all-encompassing strategy in learning environments. The significant influence of technological assistance (β0 = .27) implies that incorporating and employing instructional technology well may be especially important for promoting student engagement. Simultaneously, the noteworthy impacts of self-efficacy (β = 0.254) and teacher support (β = 0.354) highlight the ongoing significance of interpersonal relationships and personal psychological resources in fostering student engagement. Among the variables, teacher support (β = 0.354) is the strongest predictor of student engagement at the institution under investigation. This analysis offers empirical support for instructional practices that maximize student engagement by fusing technology resources with a strong teaching presence and interventions meant to boost students’ self-efficacy.

## Discussions

The present study’s findings align with prior research, which highlights the significant role of student self-efficacy in fostering student engagement. Specifically, higher levels of self-efficacy have been consistently linked to more active participation in educational activities. This is consistent with the work of Sharma, JHemant Lata, and Nasa (2014) [38], who found that students with higher self-efficacy tend to engage more fully in learning tasks. Similarly, Lisady et al. (2023) and Meng & Zhang (2023) emphasize the positive correlation between self-efficacy and student engagement, suggesting that students who believe in their academic abilities are more likely to invest effort and persist in their studies [98, 99]. Furthermore, a meta-analysis conducted by Chang and Chien (2015) in higher education contexts confirms that student self-efficacy remains a critical factor in enhancing engagement, reinforcing the notion that confident learners are more engaged in their academic endeavors [100].

The current findings align with Lent and Brown (2008), who stress the vital role of self-efficacy in promoting engagement and academic success, underscoring its important impact on student persistence and performance [101]. This aligns with the broader framework of motivational theories in education, which emphasize the link between self-belief and student involvement in learning activities. Usher and Pajares (2012) further elaborate on this connection, noting that self-efficacy beliefs significantly affect motivation and academic outcomes, fostering greater engagement and persistence in learning [29]. Artino (2012) extends these findings within the context of higher education, reinforcing the idea that self-efficacy positively impacts both engagement and academic achievement [102]. Furthermore, Lent and Brown (2008) confirm that self-efficacy remains a crucial determinant of student engagement and success, particularly in higher education settings, validating the relationship between self-belief and learning involvement across diverse educational contexts [101]. The consistency of these findings across various studies underscores the importance of fostering self-efficacy to enhance engagement and improve educational outcomes in diverse academic settings [103]. Therefore, this study adds to a growing body of literature emphasizing the value of supporting students’ self-efficacy as a means to promote active and sustained engagement in their learning experiences

Numerous studies have highlighted the pivotal role of teacher support in fostering student engagement, particularly by enhancing critical thinking, autonomy, and intrinsic motivation. Guo et al. (2023) emphasize that teacher-student interactions significantly contribute to improving students’ engagement levels by providing intellectual guidance and fostering a positive learning environment [50]. Similarly, Aliabadi and Weisi (2023) explore how teachers’ active encouragement and personalized support facilitate deeper learning and engagement [51]. Moreover, Ahmed et al. (2018) argue that teacher support is not only essential for students’ academic success but also for promoting motivation and a sense of responsibility, which are critical components of engagement [48]. These studies collectively suggest that when students feel supported and valued by their instructors, they are more likely to become actively involved in their learning process, leading to enhanced academic outcomes.

Recent studies continue to highlight the critical role of teacher support in enhancing student engagement and academic outcomes. For instance, a meta-analysis by Karabenick and Sharma (1994), later expanded in 2023, shows a significant positive relationship between teacher support and students’ academic emotions, including increased motivation and reduced anxiety, which boosts both emotional and cognitive engagement [104]. Further supporting this, research by Huang and Wang (2023) underscores that teacher support in online learning environments is essential for fostering student engagement and academic self-efficacy, directly contributing to improved academic performance [49]. Given these findings, it is essential for educators to continue to prioritize the development of supportive relationships with students. By fostering a nurturing environment, educators can significantly influence students’ emotional well-being and academic success. Therefore, teacher support should be seen as a critical factor for both engagement and academic achievement, particularly in higher education and online learning environments. Future research could explore further the mechanisms through which teacher support influences student outcomes, and how these effects can be maximized in different educational settings.

The findings of this study align with existing literature that underscores the transformative role of technology support in fostering student engagement in higher education. Advanced digital tools, such as augmented reality and multimedia-based learning, have proven effective in enhancing academic outcomes by creating immersive and interactive learning experiences. These innovations accommodate diverse learning preferences, enabling students to engage flexibly with content, fostering deeper comprehension, and promoting motivation. For instance, Kakada et al. (2019) emphasize the value of digital resources like e-books and multimedia in catering to digital-native students, offering flexibility and accessibility that drive active participation [84]. Similarly, Mayer (2014) notes that interactive elements, such as quizzes and animations in e-learning materials, enhance engagement and critical thinking skills [105].

Furthermore, the integration of blended instructional approaches demonstrates significant potential in personalizing learning environments and fostering persistence. Studies by Ghanbaripour et al. (2024) and Schuetze et al. (2023) highlight the role of technology in addressing challenges related to traditional teaching methods, such as limited engagement and inclusivity [106, 107]. By supporting diverse learning needs through platforms that facilitate interactive and flexible engagement, technology bridges gaps in accessibility and inclusivity, contributing to higher academic success. These findings reinforce the necessity for educational institutions to adopt technology-enhanced strategies, particularly in a rapidly digitalizing educational landscape, to meet the evolving needs of modern learners [108].

In conclusion, the findings confirm that technology support significantly enhances student engagement by creating flexible, interactive, and inclusive learning environments. By addressing diverse learning needs and promoting active participation, advanced digital tools and blended approaches play a vital role in improving academic outcomes and ensuring broader accessibility in higher education.

This study is limited by its focus on a single institution, Mattu University, which may limit the generalizability of the findings to other Ethiopian higher education contexts. The cross-sectional design captures data at only one point in time, restricting insights into the long-term effects of self-efficacy, teacher support, and technology on student engagement. Additionally, the reliance on self-reported questionnaires may introduce biases related to respondents’ perceptions and honesty. The study also does not fully address the complex influence of external factors, such as socio-economic conditions and institutional policies, which may affect student engagement but fall outside its scope. A cross-sectional design has limitations, such as its inability to establish causality and its reliance on data collected at a single point in time, which may not capture changes or trends over time [109]. However, it is suitable for this study as it efficiently examines the current relationships between self-efficacy, teacher support, technology support, and student engagement, providing a snapshot of these dynamics within a large, diverse sample [110].

### Implications for research, practice, and policy

Teachers must prioritize building strong teacher-student relationships, as their support is a key predictor of student engagement. By fostering trust, offering constructive feedback, and creating inclusive and interactive learning environments, educators can enhance students’ motivation and resilience [48, 111]. Incorporating student-centered approaches, such as the flipped classroom model, empowers learners to take an active role in their education, fostering both self-efficacy and engagement [35, 112]. Professional development programs focusing on these strategies are crucial for adapting teaching practices to modern educational demands [49].

Educational leaders should invest in capacity-building programs that enhance teacher support, self-efficacy, and the integration of technology into teaching. Initiatives such as mentorship programs, institutional support for flipped classroom methodologies, and infrastructure development are critical for fostering an engaging learning environment [78, 84]. Leaders must also establish systems for regular monitoring and evaluation of engagement strategies to ensure continuous improvement and alignment with institutional goals efficacy [10]

Policy makers must emphasize the integration of holistic engagement frameworks that address behavioral, emotional, and cognitive aspects of learning. This includes creating policies to promote teacher training, investing in digital infrastructure, and encouraging collaborative learning approaches to build a sense of belonging and active participation collaboration [20, 22]. Long-term strategies should prioritize funding for technology-enhanced learning, national frameworks for teacher support, and evidence-based interventions to enhance self-efficacy among students [113, 114]. These actions will enable institutions like Mattu University to create transformative learning environments that align with Ethiopia’s higher education objectives.

This study paves the way for further research into the long-term effects of self-efficacy, teacher support, and technology on academic outcomes. Future studies should investigate how these factors interact with cultural and institutional variables across various Ethiopian universities [20]. Moreover, employing mixed or qualitative methods could provide deeper insights into the complexities of student engagement.

## Conclusions

This study underscores the critical importance of self-efficacy, teacher support, and technology support in enhancing student engagement within Ethiopian higher education, specifically at Mattu University. The findings reveal that these factors collectively account for a significant portion of the variance in student engagement, with teacher support emerging as the strongest predictor. This highlights the necessity for educators to cultivate supportive teacher-student relationships and adopt instructional strategies that promote active learning and intrinsic motivation. Moreover, the role of technology support cannot be underestimated, as it facilitates access to resources and fosters an engaging learning environment tailored to the needs of digitally-savvy students. The study reinforces the idea that enhancing academic self-efficacy is vital for encouraging students to participate actively in their education, suggesting that interventions aimed at boosting confidence and competence can lead to improved academic outcomes. To address the challenges of low engagement levels, higher education institutions in Ethiopia should prioritize professional development for teachers, integrate technology effectively into the curriculum, and implement strategies that promote student self-efficacy. By doing so, they can create a more engaging and supportive educational environment that not only meets the needs of contemporary learners but also contributes to the overall success and resilience of students in their academic pursuits. This study provides valuable insights and practical recommendations that can inform policies and practices aimed at improving student engagement in similar educational contexts.

## Supporting information

S1 File Tools for factors affecting student engagement. (DOCX)

S2 File Consent to participate in research. (DOCX)

S3 File Tools for assessing the level of student engagement. (DOCX)
