# Supplementary material for: Major factors influencing student engagement in Ethiopian higher education institutions: Evidence from one institution
Source: PLoS One. 2025 Feb 6;20(2):e0318731. doi: 10.1371/journal.pone.0318731 (PMC11801635; doi:10.1371/journal.pone.0318731)
Supplement: S1 File — (DOCX) [file pone.0318731.s001.docx]

**Tools for Factors Affecting Student Engagement**

**Mattu University**

**Questionnaire for Students**

We seek authentic information for our research on the factors influencing student engagement at Mattu University through these surveys. The accuracy of your responses is crucial to the reliability of our study. Therefore, we kindly request that you provide truthful information. The researcher guarantees that any information you provide will be kept confidential and used solely for the purposes of this study.

We appreciate your cooperation in advance.

**Part one: General information**

College _______________________Department____________________ Batch or year______

Sex:

Male

Female

***Note***

- No need of writing your name√
- Please mark each choice with a "√" to reflect the degree of your academic engagement based on the provided items.

**NB: 1-I completely disagree; 2- I disagree ; 3-I cannot decide; 4-I agree; 5- I completely agree**

**Student Related Factors**

| SN | Items | 1 | 2 | 3 | 4 | 5 |
| --- | --- | --- | --- | --- | --- | --- |
|  | **Self-efficacy** |  |  |  |  |  |
| 1 | I frequently ask for clarification from my teachers when something is unclear. |  |  |  |  |  |
| 2 | I have confidence in my ability to comprehend difficult course material. |  |  |  |  |  |
| 3 | With my fellow students, I usually have academic discussions. |  |  |  |  |  |
| 4 | I am confident that I can perform well in difficult exams |  |  |  |  |  |
| 5 | When my friends ask me for academic assistance, I am confident that I can help them. |  |  |  |  |  |
| 6 | I feel comfortable getting up and delivering presentations for group tasks. |  |  |  |  |  |
| 7 | When teachers ask questions in class, I usually respond. |  |  |  |  |  |
| 8 | I request assistance from my peers when I am having problems with certain subjects. |  |  |  |  |  |
| 9 | I set attainable goals by being aware of my strengths and weaknesses. |  |  |  |  |  |
| 10 | I perform at my highest level during exams. |  |  |  |  |  |
|  | **Teacher Support** |  |  |  |  |  |
| 1 | I feel at ease discussing ideas with my instructors. |  |  |  |  |  |
| 2 | Teachers give sincere, targeted praise. |  |  |  |  |  |
| 3 | Teachers communicate learning objectives clearly |  |  |  |  |  |
| 4 | Teachers offer opportunities for in-depth understanding, analysis, critical thinking, and problem solving. |  |  |  |  |  |
| 5 | Instructors integrate collaboration among learners into their lessons. |  |  |  |  |  |
| SN | Items | 1 | 2 | 3 | 4 | 5 |
| 6 | My teachers help me with complex topics. |  |  |  |  |  |
| 7 | Teachers pay attention to the opinions I have as a student. |  |  |  |  |  |
| 8 | Instructors encourage me in taking ownership of my education |  |  |  |  |  |
| 9 | Teachers value me as their student. |  |  |  |  |  |
| 9 | Instructors are passionate about what they teach. |  |  |  |  |  |
| 10 | Teachers adjust their lessons to suit the needs of their students |  |  |  |  |  |
|  | **Technological Support** |  |  |  |  |  |
| 1 | My institution has well equipped physical library |  |  |  |  |  |
| 2 | My institution has well equipped digital library |  |  |  |  |  |
| 3 | My institution provides students with adequate internet and ICT facilities. |  |  |  |  |  |
| 4 | In my college, I have access to learning materials at all times and places. |  |  |  |  |  |
| 5 | Every student has equitable access to digital resources. |  |  |  |  |  |
| 6 | Libraries and lab centers provide plenty of opportunities to use computers. |  |  |  |  |  |
| 7 | My institution's library services are adequate to satisfy my needs. |  |  |  |  |  |
