# Supplementary material for: Major factors influencing student engagement in Ethiopian higher education institutions: Evidence from one institution
Source: PLoS One. 2025 Feb 6;20(2):e0318731. doi: 10.1371/journal.pone.0318731 (PMC11801635; doi:10.1371/journal.pone.0318731)
Supplement: S2 File — (DOCX) [file pone.0318731.s002.docx]

**Consent to Participate in Research**

Project Title: “*Major Factors Influencing Student Engagement in Ethiopian Higher Education: Evidence from One Institution”*

I willingly agree to take part in this survey. I understand that the study aims to collect information about student engagement levels and the key factors that affect it. I acknowledge that the data gathered will be used for this purpose and will remain confidential. I am also aware that there are no negative consequences if I decide not to participate or answer any questions.

Notice: *If you choose to complete the questionnaires, please sign below.*

Signature of Research Participant

----------------------------------------- ---------------

Signature of participant Date
