# Supplementary material for: Major factors influencing student engagement in Ethiopian higher education institutions: Evidence from one institution
Source: PLoS One. 2025 Feb 6;20(2):e0318731. doi: 10.1371/journal.pone.0318731 (PMC11801635; doi:10.1371/journal.pone.0318731)
Supplement: S3 File — (DOCX) [file pone.0318731.s003.docx]

**Tools for assessing the level of student engagement**

**Mattu University**

**Questionnaire for Students**

We seek authentic information for our research on the factors influencing student engagement at Mattu University through these surveys. The accuracy of your responses is crucial to the reliability of our study. Therefore, we kindly request that you provide truthful information. The researcher guarantees that any information you provide will be kept confidential and used solely for the purposes of this study.

We appreciate your cooperation in advance.

**General Information**

Which college do you belong to?  _____________________________________________

Which department do you belong to?   _____________________________________________

Sex____________ Bach (Year)___________

Notes

Please mark your responses to the items below by putting a “✓” or “✗” mark in the box provided alongside the question number.

Hint: 1- Very low; 2- Low; 3- Medium; 4- High; 5- Very high

**Behavioral Engagement**

| **Indicators** | **1** | **2** | **3** | **4** | **5** |
| --- | --- | --- | --- | --- | --- |
| 1. I participate in class discussions and answer questions. |  |  |  |  |  |
| 1. I talk about what I learn in class a lot with my friends. |  |  |  |  |  |
| 1. Whenever I have any questions during class, I ask my teacher.. |  |  |  |  |  |
| 1. I participate in group projects with classmates throughout class time. |  |  |  |  |  |
| 1. I score good grade |  |  |  |  |  |
| 1. I always study persistently. |  |  |  |  |  |
| 1. I complete a project or individual tasks as directed by my teacher by deadline |  |  |  |  |  |
| 1. I frequently attend lessons. |  |  |  |  |  |
| 1. I simply pretend to be working when I’m in class |  |  |  |  |  |
| 1. I utilize other learning materials outside that my instructor has offered. |  |  |  |  |  |

**Emotional Engagement**

| Indicators | **1** | **2** | **3** | **4** | **5** |
| --- | --- | --- | --- | --- | --- |
| 1. It's interesting to be in my class. |  |  |  |  |  |
| 1. I consider myself to be a vital component of my learning team. |  |  |  |  |  |
| 1. I believe that interacting with my peers has improved my understanding |  |  |  |  |  |
| 1. I am energized by the activities we undertake in the classroom. |  |  |  |  |  |
| 1. I believe that what I am learning in my studies will benefit me personally. |  |  |  |  |  |
| 1. I devote myself to tutoring other students |  |  |  |  |  |
| 1. I collaborate with other students, and we all gain knowledge from   one another. |  |  |  |  |  |
| 1. I have confidence that I can do well in the class and learn a lot. |  |  |  |  |  |
| 1. I enjoy working on projects or tasks with my classmates |  |  |  |  |  |
| 1. I find the course material to be fascinating. |  |  |  |  |  |

**Cognitive Engagement**

| Indicators | 1 | 2 | 3 | 4 | 5 |
| --- | --- | --- | --- | --- | --- |
| 1. After class sessions, I often go through my notes and materials. |  |  |  |  |  |
| 1. I understand what I’m doing. |  |  |  |  |  |
| 1. I review and correct my assignment. |  |  |  |  |  |
| 1. I share what I've learnt in class with individuals outside of school. |  |  |  |  |  |
| 1. I review the material I don't comprehend. |  |  |  |  |  |
| 1. I try to put new concepts I've learned into my own words |  |  |  |  |  |
| 1. I frequently try to comprehend things better. |  |  |  |  |  |
| 1. I recall important course content after class |  |  |  |  |  |
| 1. As I study, I make an effort to relate the content to what I already know in order to better understand it. |  |  |  |  |  |
| 1. While I learn, I consider how the knowledge may be useful in real life. |  |  |  |  |  |
